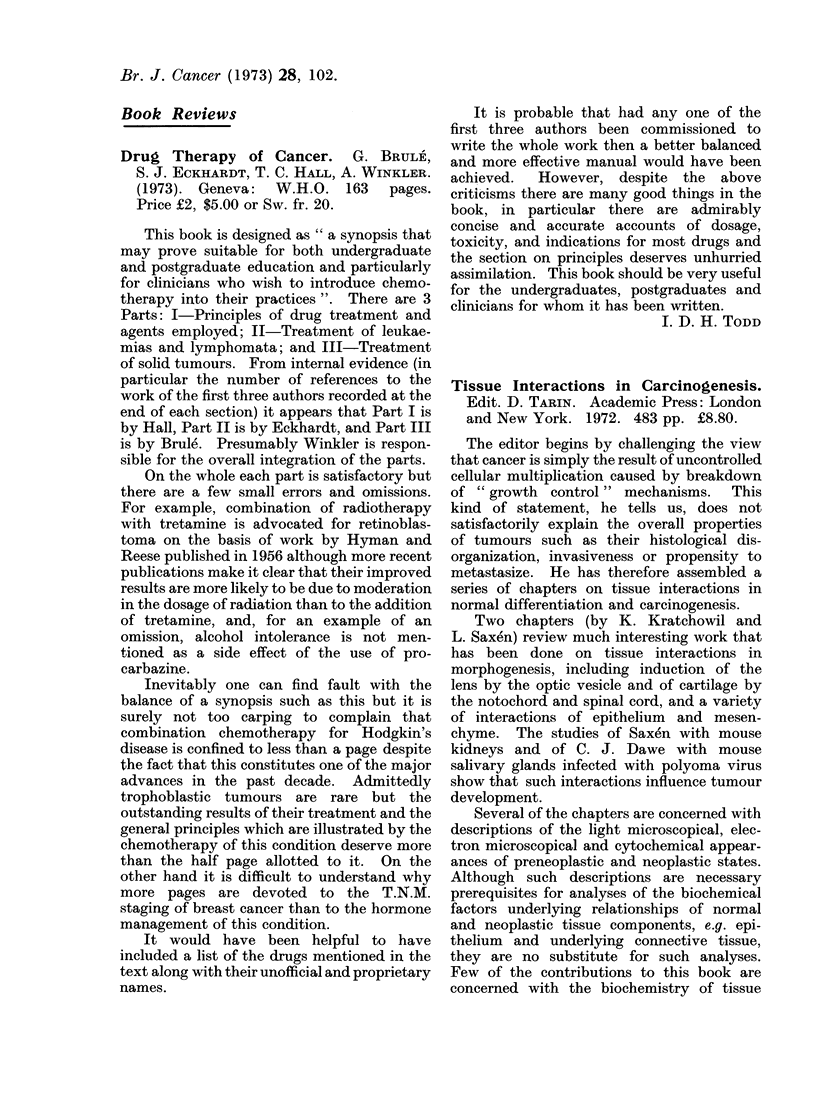# Drug Therapy of Cancer

**Published:** 1973-07

**Authors:** I. D. H. Todd


*Br. J. Cancer* (1973) **28**, 102.

### *Book Reviews*

**Drug Therapy of Cancer.** G. BRULÉ, S. J. ECKHARDT, T. C. HALL, A. WINKLER. (1973). Geneva: W.H.O. 163 pages. Price £2, $5.00 or Sw. fr. 20.

This book is designed as " a synopsis that may prove suitable for both undergraduate and postgraduate education and particularly for clinicians who wish to introduce chemotherapy into their practices ". There are 3 Parts: I—Principles of drug treatment and agents employed; II—Treatment of leukaemias and lymphomata; and III—Treatment of solid tumours. From internal evidence (in particular the number of references to the work of the first three authors recorded at the end of each section) it appears that Part I is by Hall, Part II is by Eckhardt, and Part III is by Brulé. Presumably Winkler is responsible for the overall integration of the parts.

On the whole each part is satisfactory but there are a few small errors and omissions. For example, combination of radiotherapy with tretamine is advocated for retinoblastoma on the basis of work by Hyman and Reese published in 1956 although more recent publications make it clear that their improved results are more likely to be due to moderation in the dosage of radiation than to the addition of tretamine, and, for an example of an omission, alcohol intolerance is not mentioned as a side effect of the use of procarbazine.

Inevitably one can find fault with the balance of a synopsis such as this but it is surely not too carping to complain that combination chemotherapy for Hodgkin's disease is confined to less than a page despite the fact that this constitutes one of the major advances in the past decade. Admittedly trophoblastic tumours are rare but the outstanding results of their treatment and the general principles which are illustrated by the chemotherapy of this condition deserve more than the half page allotted to it. On the other hand it is difficult to understand why more pages are devoted to the T.N.M. staging of breast cancer than to the hormone management of this condition.

It would have been helpful to have included a list of the drugs mentioned in the text along with their unofficial and proprietary names.

It is probable that had any one of the first three authors been commissioned to write the whole work then a better balanced and more effective manual would have been achieved. However, despite the above criticisms there are many good things in the book, in particular there are admirably concise and accurate accounts of dosage, toxicity, and indications for most drugs and the section on principles deserves unhurried assimilation. This book should be very useful for the undergraduates, postgraduates and clinicians for whom it has been written.

I. D. H. TODD